# Analysis of the Purpose of State Health Departments' Tweets: Information Sharing, Engagement, and Action

**DOI:** 10.2196/jmir.3002

**Published:** 2013-11-11

**Authors:** Rosemary Thackeray, Brad L Neiger, Scott H Burton, Callie R Thackeray

**Affiliations:** ^1^Brigham Young UniversityDepartment of Health ScienceProvo, UTUnited States; ^2^Brigham Young University - IdahoDepartment of Computer Science and EngineeringRexburg, IDUnited States

**Keywords:** social media, public health, engagement

## Abstract

**Background:**

Public health agencies are actively using social media, including Twitter. In the public health and nonprofit sectors, Twitter has been limited to one-way communication. Two-way, interactive communication on Twitter has the potential to enhance organizational relationships with followers and help organizations achieve their goals by increasing communication and dialog between the organization and its followers. Research shows that nonprofit organizations use Twitter for three main functions: information sharing, community building, and action.

**Objective:**

It is not known whether state health departments are using Twitter primarily for one-way information sharing or if they are trying to engage followers to build relationships and promote action. The purpose of this research was to discover what the primary function of Twitter use is among state health departments in the United States and whether this is similar to or different from nonprofit organizations.

**Methods:**

A complete list of “tweets” made by each state health department account was obtained using the Twitter application programming interface. We randomly sampled 10% of each state health department’s tweets. Four research assistants hand-coded the tweets’ primary focus (organization centric or personal health information centric) and then the subcategories of information dissemination, engagement, or action. Research assistants coded each tweet for interactivity, sophistication, and redirects to another website. Data were analyzed using SPSS version 20.

**Results:**

There were 4221 tweets from 39 state health departments. There was no statistically significant difference in the number of tweets made by a state health department and the state population density (*P*=.25). The majority of tweets focused on personal health topics (69.37%, 2928/4221) while one-third were tweets about the organization (29.14% , 1230/4221). The main function of organization-based tweets was engagement through conversations to build community (65.77%, 809/1236). These engagement-related tweets were primarily recognition of other organizations’ events (43.6%, 353/809) and giving thanks and recognition (21.4%, 173/809). Nearly all of the personal health information-centric tweets involved general public health information (92.10%, 1399/1519) and 79.03% (3336/4221) of tweets directed followers to another link for more information.

**Conclusions:**

This is the first study to assess the purpose of public health tweets among state health departments. State health departments are using Twitter as a one-way communication tool, with tweets focused primarily on personal health. A state health department Twitter account may not be the primary health information source for individuals. Therefore, state health departments should reconsider their focus on personal health tweets and envision how they can use Twitter to develop relationships with community agencies and partners. In order to realize the potential of Twitter to establish relationships and develop connections, more two-way communication and interaction are essential.

## Introduction

 Individual and organizational use of social media is rapidly increasing. Researchers estimate that by 2017, the number of individuals and corporations who have social networking accounts will reach more than 4.8 billion [1]. In particular, Twitter is becoming ever more popular. A total of 18% of Internet users have a Twitter account [2]. Among Fortune 500 companies, 77% have Twitter accounts [3] and among Forbes’ 200 largest charities, all use at least one form of social media, with 96% using Twitter [4]. Public health agencies are actively using social media, including Twitter [5-8]. Twitter has been used to disseminate information about diabetes [9], breast cancer [10], to communicate during a disaster [11], and to understand health-related trends and issues such as influenza [12], tobacco [13], prescription drug misuse [14], and suicide [15].

Fundamentally, organizations use social media sites to build relationships [16-20]. Twitter research shows that an organization’s relationship with customers is influenced by the organization’s interactivity or level of communication and contact [21]. An organization that includes social media as part of its communication strategy has the potential to increase the level of communication dialog with its customers. For example, social media can facilitate customers talking with each other, as well as customers talking with the organization [22]. When dialog and communication occur, relationships are built and these relationships are often tied to key organizational outcomes [23]. The Twitter homepage specifically states that Twitter allows businesses to connect in real-time to their customers to share product information, gather market research data, and develop relationships with both customers and partners [24]. Organizations that have relationships with customers can use Twitter to enhance brands, increase visibility, support customers, network, communicate internally, generate leads, and support other online presences [25].

Though use of Twitter has the potential to increase communication and dialog among organizations and its followers, in the nonprofit and public health sectors it has been limited to one-way communication [7,26], meaning a message is sent from one person or organization to a receiver with no expectation of a response. However, the value of social media is its ability to create two-way, interactive communication between two or more people. This two-way communication can enhance organizational relationships with followers and help organizations achieve their goals. On Twitter, interactive communication is achieved, in part, by the use of the @ symbol (which directs a message to a specific Twitter user), the user responding to public reply messages, the user asking for feedback in a “tweet”, and the use of personal pronouns (eg, us, we, you) [27].

In a study of nonprofit organizations, Lovejoy and Saxton [28] identified three main functions for organizational Twitter use, namely: information sharing, community building, and action. Information sharing meant that the organization used Twitter to disseminate information including facts about the organization and its activities. Community building was a function served by tweets that aimed to build a community or network among followers through dialog and interactivity. Finally, the function of action was indicated by tweets that asked followers to do something for the organization. Lovejoy and Saxton found that nonprofit organizations primarily used Twitter as a one-way communication tool to convey information to their followers (58.6%); one-quarter of tweets were found to be community building and only 16% were action based. The authors concluded their study by calling for more research about how governments use social media.

This study expands the work of Lovejoy and Saxton by exploring the purposes for Twitter use among public health agencies, specifically state health departments. As reported earlier, though public health agencies use social media including Twitter, it is not known for what purpose they use Twitter and more particularly, if their purposes for using Twitter are similar to or different from nonprofit organizations. More specifically, it is not known whether state health departments are using Twitter primarily for one-way information sharing or if they are trying to engage followers to build relationships. Therefore, the purpose of this research was to answer the following two research questions: (1) What is the main function of state health department tweets—information, engagement, or action? and (2) Is the content of state health department tweets more often about the organization or about personal health?

## Methods

This study involved state health departments in the United States. A list of all state health departments was obtained from the Association of State and Territorial Health Officers (ASHTO) website [29] and from the Centers for Disease Control and Prevention website [30].

State health departments represent all 50 US states [31], with 55% of state health departments classified as freestanding or independent (ie, the agency provides public health/health services only). The other 45% are categorized as super or umbrella agencies and provide additional services related to Medicaid and other public assistance, mental health, substance abuse, environmental protection, aging, child and family services, and so forth [31].

State health departments are granted legislative authority through codes and statutes to promote and protect public health and safety. These responsibilities are usually addressed by planning and implementing health promotion programs, enacting and enforcing laws and regulations, and providing access to primary care health services. State health departments also provide technical assistance to local health departments and nongovernmental agencies [31].

The existence of each state health department Twitter account was verified by three means: (1) visiting the state health department home page to ascertain the presence of a Twitter icon, (2) searching on Twitter for the name of the state health department, and (3) performing a Google search for the state health department Twitter account. A complete list of tweets made by each state health department account was obtained using the Twitter application programming interface (API) during July 2012. Because the Twitter API limits the maximum number of tweets that can be retrieved to the most recent 3200 per account, this limit was considered a complete tweet list for any state health department account that exceeded 3200 tweets. Because the intent of this study was to analyze state health departments collectively, not individually, we randomly sampled 10% of each state health department’s tweets.

We created a coding instrument based on Lovejoy and Saxton’s original classifications [28]. Their coding classifications related exclusively to organization-focused tweets [28]. We expanded the classification by adding a second category that included a personal health information focus. This decision was made based on two factors. First, an initial analysis of the tweets showed there were several public health information-only tweets, and second, results from a related study showed personal health information tweets were common among public health agencies [8]. We recognize that at a basic level all tweets are focused on the organization and fulfilling its mission. However, for the purpose of this study, we identified organization-centric tweets as those with the purpose of building and strengthening the organization. In contrast, personal health information-centric tweets were focused on one-way information dissemination about health information.

Four research assistants hand-coded all tweets. Research assistants compared coding results and resolved any discrepancies. Discrepancies were resolved by discussing the issue and coming to a consensus. Discrepancies most often occurred because of a simple error related to data entry or a misinterpretation of the tweet.

The first step was to code each tweet according to its primary focus, whether it was an organization-centric tweet or a personal health information-centric tweet. Next, research assistants coded each organization-centric tweet to determine whether the primary function was information dissemination, engagement, or action. Information dissemination was defined as one-way sharing of information about the organization and its activities [28], and included events or services, news, facts, reports, or job announcements. Engagement tweets were posts that focused on building relationships and networks with followers [28]. These tweets included giving thanks and recognition for doing something for the organization; acknowledging another organization’s events; responding to public reply messages; asking for a response or feedback; and asking for a follow, to become a fan, or to spread the word by retweeting the message. Action-based tweets represented those that encouraged the follower to do something for the organization [28], such as inviting followers to attend events or meetings, complete a survey, donate goods or money, volunteer time, or to participate in lobbying or advocacy.

Research assistants then coded each personal health information-centric tweet as one of two subcategories: information or action. Information-based tweets were one of three types: general public health information (eg, flooding can introduce impurities to both public and private drinking water sources), risk communication (eg, disease outbreaks or natural or man-made disasters), or public health reports (eg, Injury Prevention Policy Report Shows Arkansas Making Progress, More Work Needed). Action-based tweets encouraged individuals to participate in preventive health screenings (eg, Ask your health care provider for a group B strep test when you are 35-37 weeks pregnant), modifying one’s lifestyle (eg, Portion control is a must, so keep a serving cup in your purse or briefcase for healthy meals throughout the work day), or encouraged individuals to learn more and increase their knowledge (eg, Does your child walk to school? Learn how you can help ensure safe walking routes in your community). Each classification category was mutually exclusive.

In addition, research assistants coded each tweet for the degree to which it was considered interactive, its level of sophistication, and if it redirected the follower to another site for more information. Interactivity was determined by the presence of (1) an @ reply symbol, signifying that the state health department was responding to a post made by another Twitter follower, (2) an @username, indicating that the state health department was directing its post to a specific user, and (3) the use of personal pronouns. Tweet sophistication was noted by whether or not it (1) was a truncated tweet, meaning that the state health department had posted the information on one platform (eg, Facebook) and it was automatically posted to the Twitter account as well, (2) was a retweet, and (3) included hashtags. Truncated tweets and retweets denote that the state health department was not developing content specifically for Twitter but was sharing other Twitter users’ content. Hashtags, which are used to categorize tweets so users can easily follow topics posted to Twitter, are also reflective of a more advanced Twitter user. Finally, tweets that redirected followers to another source for more information signified that the state health department was using Twitter as a one-way communication tool to spread the word and link people to more information. Data were analyzed using SPSS version 20 [32].

## Results

The final sample included 4221 tweets from 39 state health departments with a Twitter account. State health departments had a mean of 2033.2 (SD 1974.9) followers and were following a mean of 414.6 (SD 725.3) other Twitter users (Table 1).

Harris and colleagues found that local health departments that serve larger populations tweet more often than those serving smaller populations [9]. Therefore, we tested to see if there was a similar difference in the frequency of state health department tweeting based on a state’s population density, which was defined as population per square mile of land area as identified in the 2010 census [33]. States were divided into three strata: low (less than 100 persons/square mile), medium (100-200 persons/square mile), and high (more than 200 persons per square mile). There were 16 states with low population density, 10 with medium, and 13 with high. The final number of tweets by population density included 1656 low (39.23%, 1656/4221), 860 medium (20.37%, 860/4221), and 1705 high (40.39%, 1705/4221). There was no statistically significant difference in the number of tweets made by a state health department and the state population density (*P*=.25). Therefore, we did no further analysis by population density.

Three-quarters (76.14%, 3214/4221) of tweets were original, meaning the state health department created and posted the content on Twitter. Only 4.45% (192/4221) of tweets were posted from another platform such as Facebook and 79.03% (3336/4221) of tweets directed followers to another link for more information. Roughly one-third (36.88%, 1557/4221) of tweets used personal pronouns. Hashtags were included 31.15% (1315/4221) of the time, while the @ symbol was used infrequently (6.99%, 295/4221).

The primary results of the study are presented in Table 2. The majority of tweets focused on personal health topics, while one-third were tweets about the organization. The main function of state health department organization-based tweets was engagement through conversations to build community. These engagement-related tweets were primarily recognition of other organization’s events and giving thanks and recognition. Rarely was the state health department using Twitter to ask for feedback or suggestions, respond to public reply messages, ask for a response to a tweet, ask for a relationship such as becoming a follower, or to disseminate information by retweeting a post.

Just over one-quarter of organization-based tweets focused on sharing one-way information about the organization. These organization information-based tweets centered on events or services, job announcements, and facts. Only 6.67% (82/1230) of organization-based tweets related to asking followers to take action for the organization.

Personal health information-centric tweets were split nearly in half between information and action. Nearly all of the information-related tweets involved general public health information. Action-based tweets predominately encouraged followers to take action to learn more, followed by encouragement to take action to modify their lifestyle.

**Table 1:** Characteristics of state health department Twitter accounts.

State	Total tweets	Following	Followers	Date created
Alabama	1422	30	292	6/24/2011
Alaska	2029	130	2597	1/31/2009
Arizona	3199	162	4753	10/24/2008
Arkansas	677	338	1079	2/5/2010
California	2003	185	6612	4/21/2009
Colorado	348	530	812	7/7/2011
Connecticut	3199	4295	4361	4/27/2009
Delaware	2953	585	2070	6/15/2009
Florida	1164	830	1293	5/11/2011
Georgia	1544	684	710	6/27/2011
Hawaii	1604	928	1969	9/29/2009
Idaho	372	143	134	6/16/2011
Illinois	1707	526	591	9/4/2009
Indiana	143	71	87	5/18/2012
Iowa	1148	16	4535	4/30/2009
Kansas	1547	262	1370	9/1/2009
Kentucky	84	3	1643	4/28/2009
Louisiana	1030	1607	1915	8/10/2010
Maryland	983	437	1058	6/5/2009
Massachusetts	882	368	9546	3/11/2009
Michigan	823	1091	3820	7/16/2009
Minnesota	714	252	3830	3/18/2009
Mississippi	565	25	2719	9/2/2008
Missouri	1378	338	1440	9/23/2009
Nebraska	1819	664	1289	8/14/2009
New Hampshire	272	36	441	3/23/2009
New Jersey	198	18	546	2/14/2011
New Mexico	80	0	35	7/30/2010
New York	731	151	1064	3/17/2010
Ohio	668	331	1941	11/16/2009
Oklahoma	100	26	121	1/3/2012
Rhode Island	711	111	3128	4/25/2009
South Carolina	227	7	956	10/29/2010
Tennessee	2657	94	1822	10/23/2009
Utah	359	239	2853	4/28/2009
Vermont	795	99	1315	4/27/2009
Virginia	257	224	511	9/8/2010
Washington	1391	128	3692	7/23/2009
Wisconsin	436	209	347	5/4/2011

**Table 2:** State health departments’ use of Twitter.

Tweet category	Tweet subcategory		n (%)
**Organization-centric**			1230/4221 (29.14%)
	**Engagement to build community**		809/1236 (65.77%)
		Recognition of other organization’s events	353/809 (43.63%)
		Giving thanks and recognition	173/809 (21.38%)
		Ask for feedback or suggestions	28/809 (3.46%)
		Respond to public reply messages	67/809 (8.28%)
		Ask for response to a tweet	32/809 (3.96%)
		Ask for a relationship	29/809 (3.58%)
		Retweet a post	27/809 (3.34%)
		Other	100/809 (12.36%)
	**Information about the organization**		338/1230 (27.48%)
		Events or services	141/338 (41.72%)
		Job announcements	77/338 (22.78%)
		Facts	50/338 (14.79%)
		News	26/338 (7.69%)
		Reports	1/338 (0.30%)
		Other	44/338 (13.02%)
	**Action**		82/1230 (6.67%)
		Attend events	29/82 (35.37%)
		Attend meetings to provide input	6/82 (7.32%)
		Complete a survey	5/82 (6.10%)
		Donate goods or money	2/82 (2.44%)
		Volunteer time	13/82 (15.85%)
		Participate in lobbying and/or advocacy	20/82 (24.39%)
		Other	7/82 (8.54%)
**Personal health information-centric**			2928/4221 (69.37%)
	**Information**		1519/2928 (52.05%)
		Public health information	1399/1519 (92.10%)
		Risk communication	63/1519 (4.15%)
		Reports	27/1519 (1.78%)
		Other	30/1519 (1.97%)
	**Action**		1409/2926 (48.12%)
		Learn more	640/1409 (45.42%0
		Modify lifestyle	523/1409 (37.12%)
		Preventive health screenings	124/1409 (8.80%)
		Other	122/1409 (8.66%)
News			51/4221 (1.21%)
Miscellaneous			12/4221 (0.28%)

## Discussion

### Principal Findings

This study examined state health departments’ Twitter posts to determine the main function of related tweets. Results show that the majority of tweets were about personal health and a limited number were about the state health department as an organization. These results are similar to those found in a study about local health departments’ use of Twitter [8]. State health departments and other public health agencies may be unique, unlike other nonprofit organizations [28], when it comes to the main function for their social media use. State health departments do not appear to be using Twitter to build their organization and develop relationships with followers, but rather to disseminate health information.

Personal health information-centric tweets contained general public health information. This is similar to what was found among local health departments [8]. These results are comparable to a study in Australia that found government tweets were dominated by public health advice and nonspecific health conditions [34]. The predominant use of Twitter to share personal health information raises two primary questions: (1) Who are the state health departments’ Twitter followers—individuals or other organizations? and (2) If followers are individuals, do they consider the state health department to be a primary source of health information? Although people do go online seeking health information [35], this health-seeking behavior is different from being a Twitter follower of a state health department, meaning one has opted in to receive regular updates. In a study among US adults about the perceived credibility of specific health information, physicians were rated as the most credible, followed by the Internet [36]. As far as credibility of online health information, one study found that perceived credibility was generally higher when the source was a specific website [37]. Therefore, general health information on a state health department Twitter account may not be perceived as highly credible.

In using Twitter, state health departments must understand the composition of their current followers and identify who it is they are trying to reach. If the state health departments’ aim is to build a community of health-related organizations, their messages and strategy will be different than if they are aiming to attract individuals in their corresponding communities. Specifically, individual Twitter users tend to be younger, of Black or Hispanic background, have a college education, and have incomes over US$75,000 a year [2]. These may or may not be the individuals with whom the state health department is trying to cultivate relationships. Rather, state health departments may be more interested in using Twitter to develop relationships with community-based organizations and other agencies. Fostering online relationships with these agencies may result in offline collaborations. However, developing these online relationships will require concerted effort between Twitter users, as they do not appear to evolve naturally. For instance, in studying Twitter connections between state health departments, Harris and colleagues found that state health departments on Twitter tend to follow other state health departments in their region who are also on Twitter. However, the follow is not reciprocal, meaning they are not following each other back [38]. Reciprocal following builds the users network. It also allows followers to receive tweets from the other user, which are then more likely to be retweeted [39]. Additionally, social network analysis states that reciprocity indicates stronger ties among people [40]. Also, among individuals, the number of Twitter followers is linked to increased social capital [41]. There may also be a similar increase in strength of connections and social capital among connected state health departments and their followers.

The rate of tweeting information about the organization is substantially less than what was found by Lovejoy and Saxton [28] and among local health departments [8]. State health departments may be less concerned with promoting themselves as an agency and more focused on fulfilling one of public health’s ten essential services: inform, educate, and empower [42]. However, state health departments may want to reconsider how they use Twitter and create ways to convey information about the organization in order to increase the community’s awareness of the state health department, its purpose, priorities, and contribution to the state.

State health departments should continue to post engagement-related tweets that focus on recognizing other organizations’ events or giving thanks and recognition. The rate of engagement-related tweets among state health departments was more than that found by Lovejoy and Saxton for nonprofit organizations [28] and among local health departments [8]. This indicates that state health departments are making an effort to reach out to other community organizations, which is a positive step toward building relationships with current or potential partners. Both information sharing and engagement with other organizations and partners can be particularly beneficial for a state health department when engaging in advocacy-related efforts, which have long been a core public health strategy and an essential public health service [42]. In fact, use of social media has been identified as critical to influencing advocacy and social movements [43-45].

Interestingly, although state health departments were posting engagement-related tweets to foster and build relationships, very few state health departments were asking followers to take action to benefit the organization. Researchers have proposed an engagement hierarchy between organizations and followers that progresses from low to medium to high with high engagement characterized by followers becoming involved with the organization as either partners in fulfilling the organization’s goals or as direct recipients of the organization’s programs and services [46]. The hierarchy posits that high engagement is the culminating and defining purpose of social media use in public health settings. This suggests that state health departments may want to consider using Twitter to recruit followers and foster relationships that will benefit organizational causes and programs.

The majority of the tweets were original, meaning the state health departments are investing effort in creating unique content. These results are similar to local health departments’ use of Twitter [8]. This implies that the state health departments have identified specific content they want to convey to their followers and are not re-posting random tweets. Original content may also be more likely to draw the interest and attention of followers as it suggests that the organization is tailoring its posts to the interests and needs of its followers.

Twitter is being used as a one-way communication tool. Though state health departments are trying to engage in conversation, most of these tweets were about recognition of other agencies’ events and giving thanks. Rarely did state health departments attempt to engage followers in dialog by asking for a response, a retweet, and so forth. Furthermore, three-quarters of tweets included a link for where to go for more information. These results are similar to other research that showed a preponderance of hyperlinks included with tweets [26,47]. The emphasis on one-way communication is further evidenced by the lack of inclusion of the @ symbol in tweets, which would direct a message to a specific Twitter follower.

### Limitations

The results should be interpreted with the following limitations in mind. First, we were able to sample only public tweets. It is possible that state health departments are responding to individuals through direct messages, which are private, but there is no way to assess that without having access to individual accounts. Second, we observed only one side of the potential dialogue between state health departments and their followers. That is, we were able to study posts that the state health department made, but were not able to analyze Twitter posts that were directed to the state health department from other Twitter users. Third, this study is about a specific social media application, Twitter, and it could be that some state health departments behave very differently on other applications. For example, on social networking sites such as Facebook, there may be more two-way communication or the purposes for posts may be different from Twitter. Last, although there were four research assistants coding the data, there is still the possibility of coder subjectivity in interpreting the main purpose of the tweet.

### Conclusions

State health departments are using Twitter as a one-way communication tool, with tweets focused primarily on personal health. When tweeting about the organization, state health departments are trying to engage audiences through posts that focus on recognition of community and organizational events. There is potential for state health departments to use Twitter to develop relationships with other community agencies. To do so, state health departments should reconsider their focus on personal health tweets. To realize the potential of Twitter to establish relationships and develop connections with followers, more two-way communication and interaction are essential.

## Abbreviations

API: application programming interface
ASHTO: Association of State and Territorial Health Officers
